# Beyond the Boundaries of Disease—Significant Post-traumatic Growth in Multiple Sclerosis Patients and Caregivers

**DOI:** 10.3389/fpsyg.2022.903508

**Published:** 2022-06-23

**Authors:** Irene Gil-González, María Ángeles Pérez-San-Gregorio, Rupert Conrad, Agustín Martín-Rodríguez

**Affiliations:** ^1^Department of Personality, Assessment, and Psychological Treatment, Faculty of Psychology, University of Seville, Seville, Spain; ^2^Department of Psychosomatic Medicine and Psychotherapy, University Hospital Bonn, Bonn, Germany; ^3^Department of Psychosomatic Medicine and Psychotherapy, University Hospital Muenster, Muenster, Germany

**Keywords:** multiple sclerosis, post-traumatic growth, clinical variables, longitudinal, patients, caregivers

## Abstract

Despite the negative repercussions of a chronic disease, multiple sclerosis (MS) might also lead to positive consequences. This longitudinal study explored post-traumatic growth in MS patients and attempted to identify possible determinants. Post-traumatic growth of 260 patients and their caregivers was compared. A subset of 209 patients and caregivers were evaluated at baseline. Patients filled in the Posttraumatic Growth Inventory and General Health Questionnaire at three different times over a 36-month follow-up period. Patient post-traumatic growth significantly increased over the follow-up period (*p* < 0.001) with large effect sizes on almost every subscale. Higher score on the Expanded Disability Status Scale, higher pain severity, female gender, and higher anxiety were positive predictors of post-traumatic growth, while more interference of pain, higher level of education, and more social dysfunction were negative predictors. Post-traumatic growth did not differ significantly between patients and caregivers. Our results showed significant positive intrapsychic changes of MS patients over a 36-month follow-up period up to 12 years from diagnosis. The potential influence of clinical, demographic, and mental health variables underlines the need for a personalized approach to be able to understand and sustain these processes. Comparable post-traumatic growth levels in patient-caregiver dyads at baseline suggest interdependently driven cognitive processes stabilizing well-being. Future research is recommended for further insight into the underlying cognitive processes.

## Introduction

Multiple sclerosis (MS) is a chronic disease affecting physical, mental, and social well-being (Gil-González et al., 2020; Faraclas et al., 2022). While struggling with the unpredictability and variability of symptoms, MS patients may also undergo intrapsychic processes leading to positive mental and physical changes that impact on all areas of life. MS patients usually report greater appreciation of life, sense of liberation, taking better care of themselves and strengthening of close relationships (Pakenham, 2005a, 2007; Kivi et al., 2019; Baka et al., 2021). The term post-traumatic growth coined by Tedeschi and Calhoun (2004) is defined as “an experience of positive change that occurs as a result of the struggle with highly challenging life crises.” “Post-traumatic” highlights that growth is experienced after a critical life event, not just minor stressful episodes. “Growth” refers to inner development of capabilities and functioning due to modifications in mental and emotional awareness following adverse experience (Kivi et al., 2019). Post-traumatic growth must be differentiated from other, perhaps overlapping, similar concepts. For instance, resilience is the ability to adapt to change and uncertainty after an adverse and stressful situation. This flexibility prevents people from experiencing the full blow of a critical life event, consequently experiencing less trauma, and thereby, limiting post-traumatic growth. The main difference between post-traumatic growth and resilience is that while resilience permits the person to survive and adapt to the adverse situation without significant difficulties, in post-traumatic growth, a stress-induced positive change leads the person to function better than before the traumatic experience. Thus, it has been demonstrated that MS patients with a higher level of resilience experience less post-traumatic growth (Younesi et al., 2020).

Post-traumatic growth and psychological adjustment to MS are closely related, as both concepts imply regaining functionality and quality of life. However, post-traumatic growth means a level of functioning surpassing the level previous to the critical life event.

Irvine et al. (2009) identified the following topics related to potentially traumatic events and highly stressful situations for MS patients: reaction to and impact of the diagnosis; limitations in social activities due to impairments; role in society and self-worth; changes in relationship dynamics and dependency, particularly when careers are involved; attitudes and reactions of others to MS patients who feel that unaffected people cannot understand it. Thus, the extent to which the diagnosis itself or its impact on different areas of life is a major traumatic experience is highly individual.

It is evident that those mechanisms leading to significant post-traumatic growth have not been fully understood. We try to understand these mechanisms by looking at specific factors, which may contribute to this development. The identification of all relevant factors, their specific contribution as well as interdependence enable us to fully understand the underlying mechanisms of post-traumatic growth.

Post-traumatic growth has been widely explored in various medical conditions, for example, acquired brain damage (Rogan et al., 2013), cardiac outpatient (Leung et al., 2010), myocardial infarction (Rahimi et al., 2016; Hosseini Golafshani et al., 2021), cancer (Heidarzadeh et al., 2014), and liver transplantation (Pérez-San-Gregorio et al., 2017a). There is evidence that most MS patients undergo some degree of post-traumatic growth (Aflakseir and Manafi, 2018; Younesi et al., 2020), even though the underlying mechanisms are poorly understood. Pakenham and Cox (2009) discovered a significant connection between duration of MS and personal growth, but only in patients who had been diagnosed over 15 years before. The majority of studies showed controversial associations between sociodemographic or clinical variables and post-traumatic growth (Hart et al., 2008; Ackroyd et al., 2011; Heidarzadeh et al., 2014). Both younger and older age have been found to be positively related to post-traumatic growth (Leung et al., 2010; Heidarzadeh et al., 2014; Henson et al., 2021). Analysis of level of education has also shown inconsistent results. Some studies have found a positive correlation between post-traumatic growth and education, probably because it provides access to more resources for such development. However, others have found an association with a lower level of education, where intense search for social support is a key factor in post-traumatic growth (Henson et al., 2021).

In mental health, post-traumatic growth has shown a positive relationship with anxiety and negative with depressive symptoms (Barskova and Oesterreich, 2009). It might be argued that in depression, the ability for inner growth is hampered by a lack of energy and inner drive. Therefore, some psychological treatments have increased post-traumatic growth by reducing demoralization and depressive symptoms (Kivi et al., 2019) as well as amplifying positive affects (Hart et al., 2008). Other studies have not found any significant association between mental health and post-traumatic growth in MS (Ackroyd et al., 2011).

As MS is a degenerative and uncurable neurological disorder with onset most often in young adulthood, it has a strong impact on patients’ families and relatives, particularly on primary caregivers. Many studies have focused on caregiver distress in coming to terms with the repercussions of the disease (Pakenham et al., 2012; Bassi et al., 2016; Tramonti et al., 2019). However, there is also qualitative and quantitative research indicating that care of MS patients may also lead to positive experiences (Rajachandrakumar and Finlayson, 2021). In adapting to their new role, caregivers might become aware of personal strengths and family resources, or make healthy modifications in their lifestyle (Maguire and Maguire, 2020). In this context the importance of the patient-caregiver dyad, especially coping strategies, has been emphasized. Dyads using avoidance coping have shown less growth than those dealing with MS in an active and mutually supportive manner (Wawrziczny et al., 2021). Couples in which one partner is an MS patient who attended therapeutic programs where they had the opportunity to share and reevaluate their experience, showed greater post-traumatic growth (Neate et al., 2019).

Thus, benefits in patients and caregivers are strongly related. Nonetheless, care recipients typically report higher levels of post-traumatic growth. It has been suggested that the direct personal experience of a traumatic event has a particularly high psychological impact challenging one’s whole personality. This might lead to new self-definition and deeper meaning (Pakenham, 2005b; Ackroyd et al., 2011; Treder-Rochna, 2020).

Taking into account the relevance of post-traumatic growth in clinical practice and controversial knowledge about contributing factors and underlying mechanisms in MS, the present study aimed to examine post-traumatic growth in adults with MS and any changes recorded at three different times, explore possible clinical determinants of post-traumatic growth and compare the post-traumatic growth level in patients and caregivers. Against the backdrop of empirical findings, we hypothesized first, that post-traumatic growth would increase significantly over the course of 36 months (T1–T3), second, that longer duration of the disease would predict more post-traumatic growth, and third, there would be significantly higher post-traumatic growth in patients than caregivers.

## Materials and Methods

### Sample and Procedure

This prospective longitudinal observational study was done with a probability-based survey sample of MS patients. The baseline assessment (T1) took place from June 2017 to May 2018. The first follow-up after 18 months was from December 2018 to December 2019 (T2). The second follow-up after 36 months took place from May 2020 to April 2021 (T3). None of the participants received psychological treatment during the study. The caregivers of a group of 209 MS patients evaluated at T1 were also assessed at that time. The caregiver relationship to patients was: partner (64.6%), parent (17.2%), child (9.1%), sibling (6.2%), other (2.9%).

A total of 260 outpatients at the Virgen Macarena University Hospital in Seville (Spain), with a mean age of 45.1 (SD = 10.6) and 68.8% of whom were women, participated in all three stages of the study. The dyadic sample was composed of 209 patients and their caregivers with a mean age of 47.5 (SD = 3.4) and 45.4 (SD = 11.8) respectively. Sample characteristics are presented in Table 1.

**Table 1:** Clinical and sociodemographic characteristics of the study sample at T1.

	First and second objectives	Third objective	
	Patients *N* = 260	Patients *N* = 209	Caregivers *N* = 209
Gender *n* (%)			
Male	81 (31.22)	66 (31.63)	98 (46.92)
Female	179 (68.78)	143 (68.37)	111 (53.08)
Age *M* (*SD*)	45.1 (10.59)	45.4 (11.82)	47.5 (13.44)
Partnership *n* (%)			
No partner	67 (25.83)	39 (18.74)	32 (15.34)
Partner	193 (74.17)	170 (81.26)	177 (84.66)
Occupation *n* (%)			
Employed/Studying	92 (35.41)	62 (29.57)	127 (60.81)
Unemployed	168 (64.59)	147 (70.43)	82 (39.19)
Education *n* (%)			
Primary education	35 (13.52)	43 (20.63)	54 (25.87)
Secondary education	82 (31.48)	69 (33)	68 (32.51)
University or higher	143 (55)	97 (46.37)	87 (41.62)
EDSS *M* (*SD*)	3.2 (1.92)	3.7 (3.67)	
MS subtype *n* (%)			
Remittent	228 (87.74)	167 (79.9)	
Progressive	32 (12.26)	42 (20.1)	
Months since diagnosis *M* (SD)	144.8 (89.26)	148.9 (91.84)	
Months since outbreak *M* (SD)	185.8 (110.81)	187.8 (113.32)	

**Figure 1:**
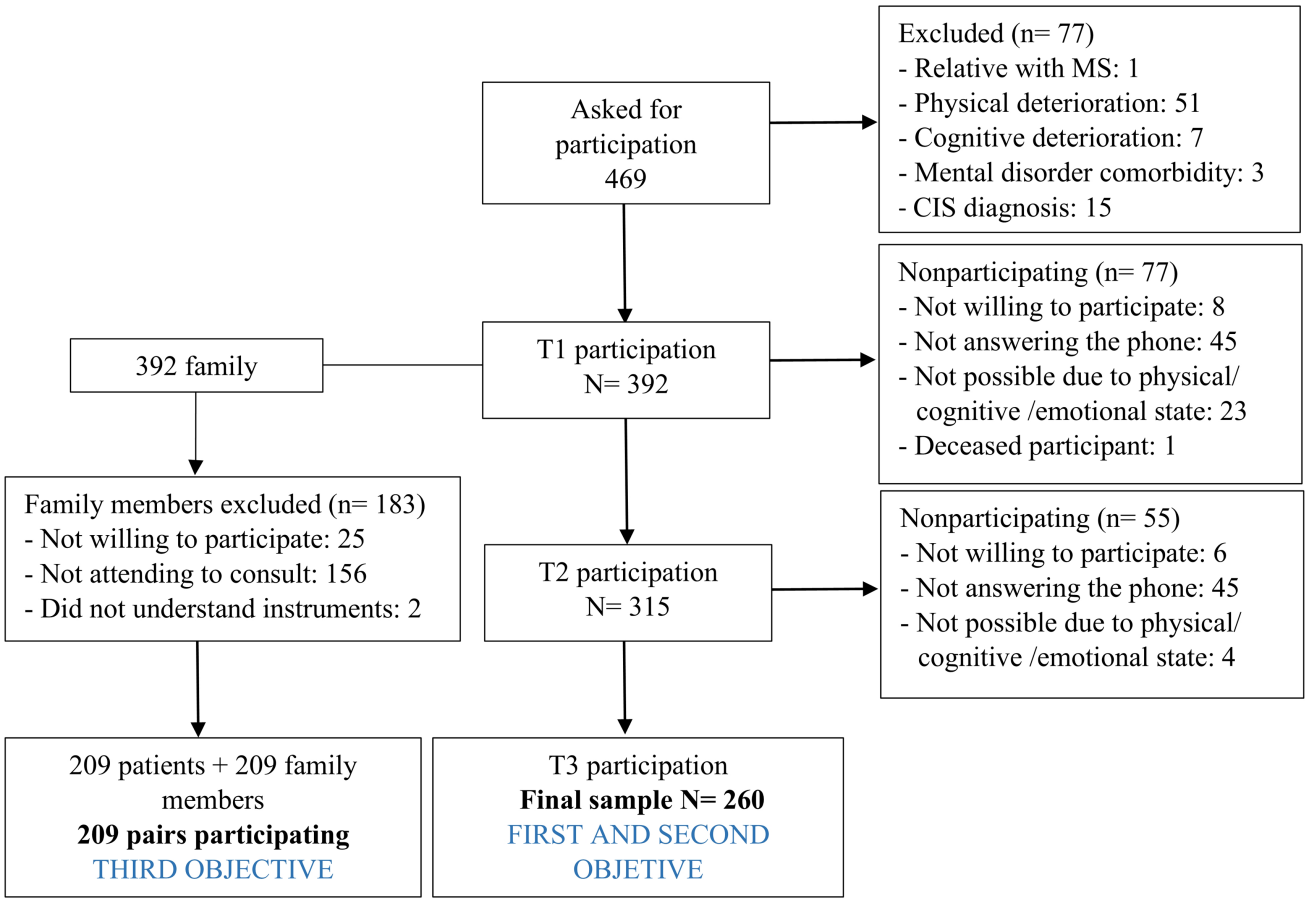
Study flowchart. CIS, Clinically isolated syndrome.

Participants met the following inclusion criteria: (1) aged 18 or over; and (2) mental, physical and cognitive ability to sign the informed consent and answer the questionnaires. Exclusion criteria were a relative with MS and MS diagnosis unconfirmed. Figure 1 summarizes the sample selection process.

After a neurological consultation at the hospital, patients and caregivers were invited to participate in the study. Participants were given written and oral information and instructions. Before participating, they received an information sheet which included the study objectives, informed consent, study benefits and risks, planned publication of results, data protection procedures, potential financial interest and researcher contact information. After patients’ questions had been answered, they signed the informed consent form, which included the study title and a written reminder of their right to end their participation at any time without any repercussions. When the informed consent had been signed, patients and caregivers answered the questionnaires. They were free to complete the questionnaires in the same or separate rooms, and a researcher was available for help if needed while they were filling them in.

The study was approved by the responsible Ethics Committee (0846-N-18).

### Instruments

Sociodemographic data were collected using a standardized questionnaire designed *ad hoc* for the study. Participants were asked their age, gender, marital status, current occupation, and education. Caregivers were asked to fill in the same questionnaire, and to state their relationship to the patient.

Clinical and diagnostic information was acquired from the medical record database.

#### Post-traumatic Growth

The Spanish version of the Post-Traumatic Growth Inventory (PTGI-21) was applied to evaluate patients’ and caregivers’ perception of their personal benefit from their experience with MS (Tedeschi and Calhoun, 1996; Weiss and Berger, 2006). The PTGI-21 contains 21 items scored on Likert scales from 0 (“no change”) to 5 (“very great degree of change”). Test results provide a total score and the following five subscales: Relating to others, new possibilities, Personal strength, Spiritual change, and Appreciation of life. Results of the Spiritual change subscale, which assesses the search for spiritual meaning of things, the quest for meaning in life and existential personal approaches, are especially important. Recent meta-regression modeling has shown Cronbach’s alpha coefficients and related confidence intervals ranged from excellent (PTGI-21 total) to good (Relating to others, New possibilities, Personal strength, Spiritual change) and acceptable (Appreciation of life) and were unrelated to patient characteristics (Lenz et al., 2021). The Cronbach’s alpha in our study was 0.92, 0.91, and 0.93 for the total score scale and 0.74–0.80, 0.77–0.83, and 0.72–0.88 for the five subscales, at T1, T2, and T3, respectively. For caregivers, the Cronbach’s alpha was 0.94 for the total scale and 0.77–0.88 for the subscales.

#### Mental Health

The General Health Questionnaire (GHQ-28) consists of 28 items on a 4-point Likert scale. The four subscales are somatic symptoms, anxiety/insomnia, social dysfunction, and depression. Subscales score from 0 to 21 and the total GHQ-28 score from 0 to 84. Higher scores indicate worse mental health (Goldberg et al., 1997; Willmott et al., 2004). The Spanish version has shown acceptable validity (Lobo et al., 1986) and reliability in studies on chronic medical conditions (Vallejo et al., 2014). In the present sample Cronbach’s alpha ranged from 0.85 to 0.94 at T1, from 0.87 to 0.92 at T2, and from 0.86 to 0.96 at T3.

### Impact of COVID

The third evaluation (T3) took place at the onset of the COVID pandemic. Participants were asked if they felt affected by the COVID situation (yes/no). Based on their answers, patients were categorized into two groups.

### Data Analysis

Descriptive analyses (means, standard deviations, and frequencies) were performed to describe clinical and sociodemographic characteristics.

A one-way ANOVA for repeated measures was applied to study longitudinal changes in patient post-traumatic growth at T1, T2, and T3.

To identify predictors of post-traumatic growth linear regression analyses were applied. Three different multivariate linear regression models were built with the total PTGI-21 score at T1, T2 and T3 as dependent variables. Demographic variables (gender, age, and education) at T1 and clinical variables (Expanded Disability Status Scale (EDSS), MS subtype, months since diagnosis, months since onset, pain severity and pain interference), and mental health outcomes (GHQ-28 subscales) at T1, T2, and T3 were the predictors.

Unpaired t-tests were used to examine mean differences in the PTGI-21 total score and subscales between patients and caregivers. Any mean differences on the PTGI-21 in patients affected versus unaffected by COVID-19 were also examined.

All statistics were computed using SPSS version 26. For all tests, level of significance was set to *p* < 0.05. The effect size was computed using G^*^Power Software and interpreted according to Cohen’s (1988) guidelines as follows: *f* (0.10 = small, 0.25 = medium, and 0.40 = large), *f ^2^* (0.02 = small, 0.15 = medium, and 0.35 = large effects) and *d* (0.20 = small, 0.50 = medium, and 0.80 = large effects).

## Results

**Figure 2:**
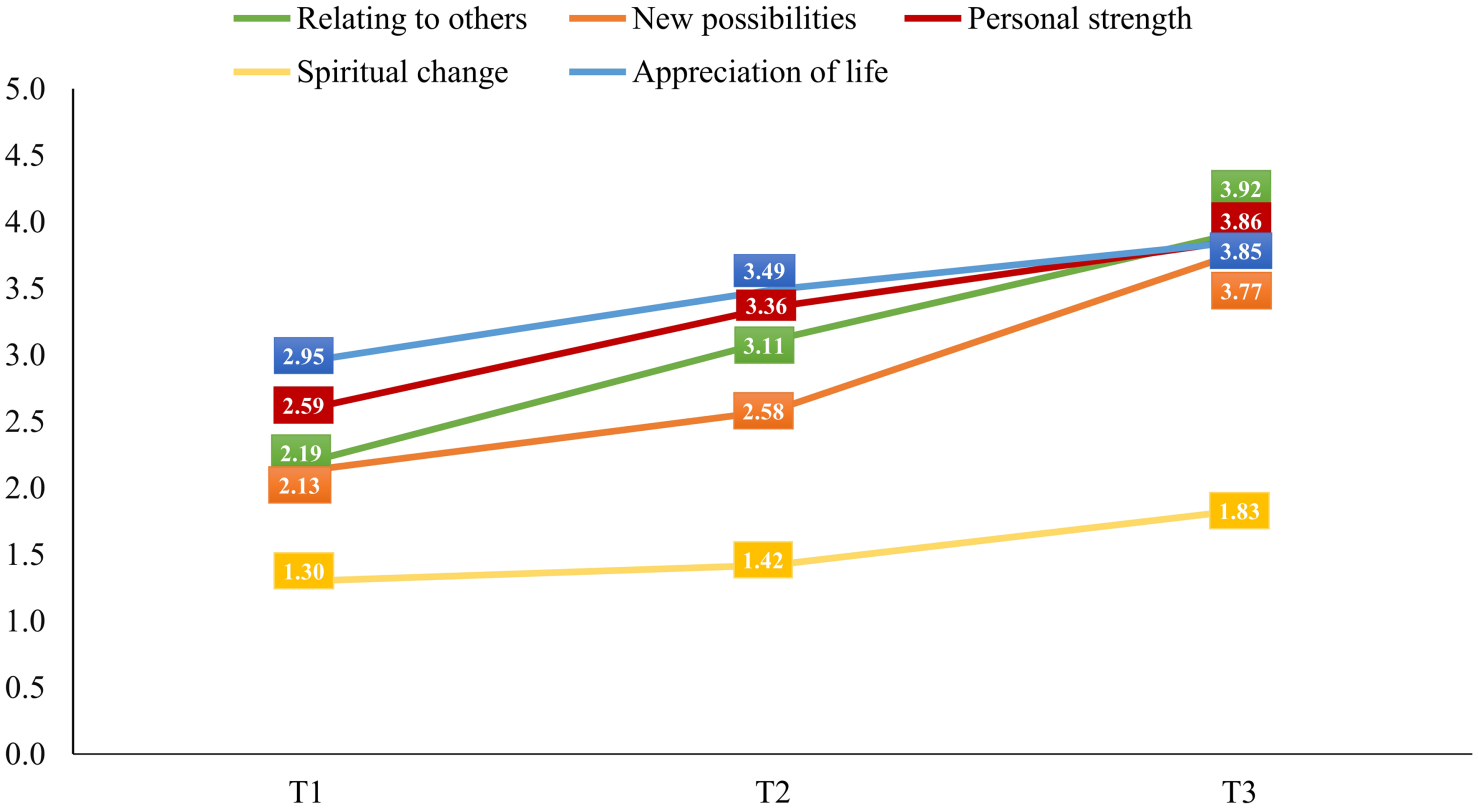
Evolution of patient post-traumatic growth over the 36-month follow-up.

A one-way ANOVA was applied to analyze differences in patient PTGI-21 mean scores at T1, T2 and T3. Mean and standard deviations are presented in Table 2. Time was significant for all PGI subscales: Relating to Others [*F* (2, 518) = 214.997, *p* < 0.001, *ƞ*^2^ = 0.455], New Possibilities [*F* (2, 518) = 189.533, *p* < 0.001, *ƞ*^2^ = 0.423], Personal Strength [*F* (2, 518) = 110.740, *p* < 0.001, *ƞ*^2^ = 0.300], Spiritual Change [*F* (2, 518) = 12.705, *p* < 0.001, *ƞ*^2^ = 0.047], Appreciation of Life [*F* (2, 518) = 47.152, *p* < 0.001, *ƞ*^2^ = 0.154], and total post-traumatic growth [*F* (2, 518) = 121.692, *p* < 0.001, *ƞ*^2^ = 0.451]. Effect sizes coefficients were large (*f* 0.427 to 0.913). Only spiritual change (*f* = 0.222) showed a small effect size. The Bonferroni correction was applied to all repeated comparisons. Post-hoc comparisons showed significant differences between T1 and T2 (*p* < 0.001 for all subscales), T1 and T3 (*p* < 0.001 for all subscales), and T2 and T3 (*p* < 0.001 for Relating to others, New possibilities, Personal strength, and *p* = 0.001 for Appreciation of Life), except for spiritual change with a non-significant difference between T1 and T2 (*p* = 0.677). Figure 2 shows PTGI-21 subscale scores over the 36-month follow-up period. There were no significant differences on any of the PTGI-21 scales between patients with remittent or progressive MS at T1, T2 and T3.

**Table 2:** Comparison of post-traumatic growth (PTGI-21) at T1, T2 and T3.

	*M* (*SD*)			Comparison over time							
	T1	T2	T3	T1-T2 (1)	T1-T3 (2)	T2-T3 (3)	Cohen’s *d*			*F* (2, 518)	Cohen’s *f*
							1	2	3		
Relating to others	2.19 (1.32)	3.11 (1.20)	3.92 (0.96)	*p* < 0.001	*p* < 0.001	*p* < 0.001	−1.322 (L)	−2.411 (L)	−1.352 (L)	214.997^**^	0.913 (L)
New possibilities	2.13 (1.31)	2.58 (1.29)	3.77 (1.08)	*p* < 0.001	*p* < 0.001	*p* < 0.001	−0.645 (M)	−2.264 (L)	−1.763 (L)	189.533^**^	0.856 (L)
Personal Strength	2.59 (1.39)	3.36 (1.20)	3.86 (1.06)	*p* < 0.001	*p* < 0.001	*p* < 0.001	−1.089 (L)	−1.672 (L)	−0.834 (L)	110.740^**^	0.655 (L)
Spiritual change	1.30 (1.49)	1.30 (1.67)	1.83 (1.91)	*p* = 0.677	*p* < 0.001	*p* = 0.001	−0.105 (N)	−0.562 (M)	−0.446 (S)	12.705^**^	0.222 (S)
Appreciation of life	2.95 (1.56)	3.49 (1.49)	3.85 (1.24)	*p* < 0.001	*p* < 0.001	*p* = 0.001	−0.755 (M)	−1.140 (L)	−0.474 (S)	47.152^**^	0.427 (L)
Total PTG	47.83 (23.63)	61.50 (21.72)	76.95 (19.49)	*p* < 0.001	*p* < 0.001	*p* < 0.001	−1.212 (L)	−2.345 (L)	−1.496 (L)	121.692^**^	0.906 (L)

L, Large effect size; M, Medium effect size; S, Small effect size; N, Null effect size; PTG, Post-traumatic growth.

** *p* < 0.01.

The influence of patients’ clinical, demographic, and mental health variables on their total post-traumatic growth score was analyzed. The three multivariate regression models are presented in Table 3. In Model 1 [*F* (14,245) = 1.956, *R*^2^ = 0.096, *p* = 0.032], social dysfunction (*β* = −0.214, *p* = 0.010) and anxiety/insomnia (*β* = 0.209, *p* = 0.028) were significant negative and positive predictors of PGI-total score at T1, respectively. All variables included in Model 1 explained 9.6% of PGI-21 total score variance at T1, with a small effect size (*f ^2^ =* 0.106).

**Table 3:** T1, T2 and T3 Post-traumatic growth (PTGI-21) multiple linear regression model.

	*Dependent variable T1 post-traumatic growth (PGI-21)*						
	*F* (14,245)	*R* ^2^	*B*	*p*	*SE.B*	*β*	*f* ^2^
**Model 1**	1.956, *p* = 0.032	0.096	67.413		11.01		0.106
Gender			3.134	0.330	3.213	0.062	
Age			−0.319	0.069	0.174	−0.143	
Education			−3.881	0.068	2.115	−0.118	
EDSS			1.858	0.042	0.911	0.152	
MS type			0.018	0.997	5.093	0.000	
DMD type			−1.042	0.439	1.343	−0.051	
Months since diagnosis			0.013	0.662	0.029	0.048	
Months since outbreak			−0.005	0.856	0.025	−0.021	
Pain severity			0.914	0.387	1.056	0.101	
Pain interference			−0.919	0.319	0.920	−0.122	
Somatic symptoms			0.107	0.823	0.477	0.021	
Anxiety and insomnia			0.925	0.028	0.419	0.209	
Social dysfunction			−1.370	0.010	0.530	−0.214	
Severe depression			−0.529	0.226	0.436	−0.102	
***Dependent variable T2 post-traumatic growth (PGI-21)***							
**Model 2**	2.693, *p* = 0.001	0.133	74.745		11.259		0.153
Gender			6.849	0.022	2.971	0.146	
Age			−0.217	0.166	0.156	−0.106	
Education			−1.197	0.530	1.902	−0.629	
EDSS			2.007	0.025	0.888	0.018	
MS type			−1.707	0.716	4.687	−0.028	
DMD type			−0.486	0.699	1.254	−0.025	
Months since diagnosis			−0.007	0.789	0.026	−0.268	
Months since outbreak			−0.002	0.934	0.022	−0.009	
Pain severity			−0.516	0.608	1.005	−0.65	
Pain interference			1.190	0.253	1.034	0.148	
Somatic symptoms			0.022	0.960	0.447	0.005	
Anxiety and insomnia			0.424	0.253	0.370	0.100	
Social dysfunction			−2.085	<0.001	0.515	−0.367	
Severe depression			−0.386	0.378	0.437	−0.065	
***Dependent variable T3 post-traumatic growth (PGI-21)***							
**Model 3**	2.054, *p* = 0.015	0.054	85.295		10.387		0.054
Gender			4.578	0.085	2.649	0.109	
Age			−0.107	0.441	0.139	−0.058	
Education			−4.640	0.007	1.172	−0.071	
EDSS			1.309	0.096	0.783	0.135	
MS type			−4.578	0.240	3.886	−0.087	
DMD type			−0.819	0.488	1.118	−0.045	
Months since diagnosis			−0.015	0.434	0.020	−0.71	
Months since outbreak			0.018	0.208	0.014	0.114	
Pain severity			2.412	0.005	0.861	0.330	
Pain interference			−2.031	0.030	0.931	−0.271	
Somatic symptoms			−0.717	0.184	0.516	−0.132	
Anxiety and insomnia			0.386	0.568	0.290	0.100	
Social dysfunction			0.316	0.568	0.551	0.056	
Severe depression			−0.331	0.390	0.384	−0.070	

EDSS, Expanded disability status scale; MS, Multiple sclerosis; DMD, Disease modifying drug; S, Small effect size.

Model 2 [*F* (14,245) = 2.693*, R*^2^ = 0.133, *p* = 0.001] showed that female gender (*β* = 0.146, *p* = 0.022), and high EDSS (*β* = 0.018, *p* = 0.025) predicted PGI-21 total score at T2 positively and social dysfunction (*β* = −0.367, *p* < 0.001) predicted it negatively. The variables in Model 2 accounted for 13.3% of total PGI-21 score variance at T2 with a medium effect size (*f ^2^ =* 0.153).

In Model 3 [*F* (14,245) = 2.054, *R*^2^ = 0.054*, p* = 0.015] education (*β* = −0.071, *p* = 0.007) and pain interference (*β* = −0.271, *p* = 0.030) were the negative predictors of PGI-21 total score at T3 and pain severity (*β* = 0.330, *p* = 0.005) was a positive predictor. Model 3 variables explained 5.4% of PGI-21 total score variance at T3 with a small effect size (*f  ^2^ =* 0.054).

**Figure 3:**
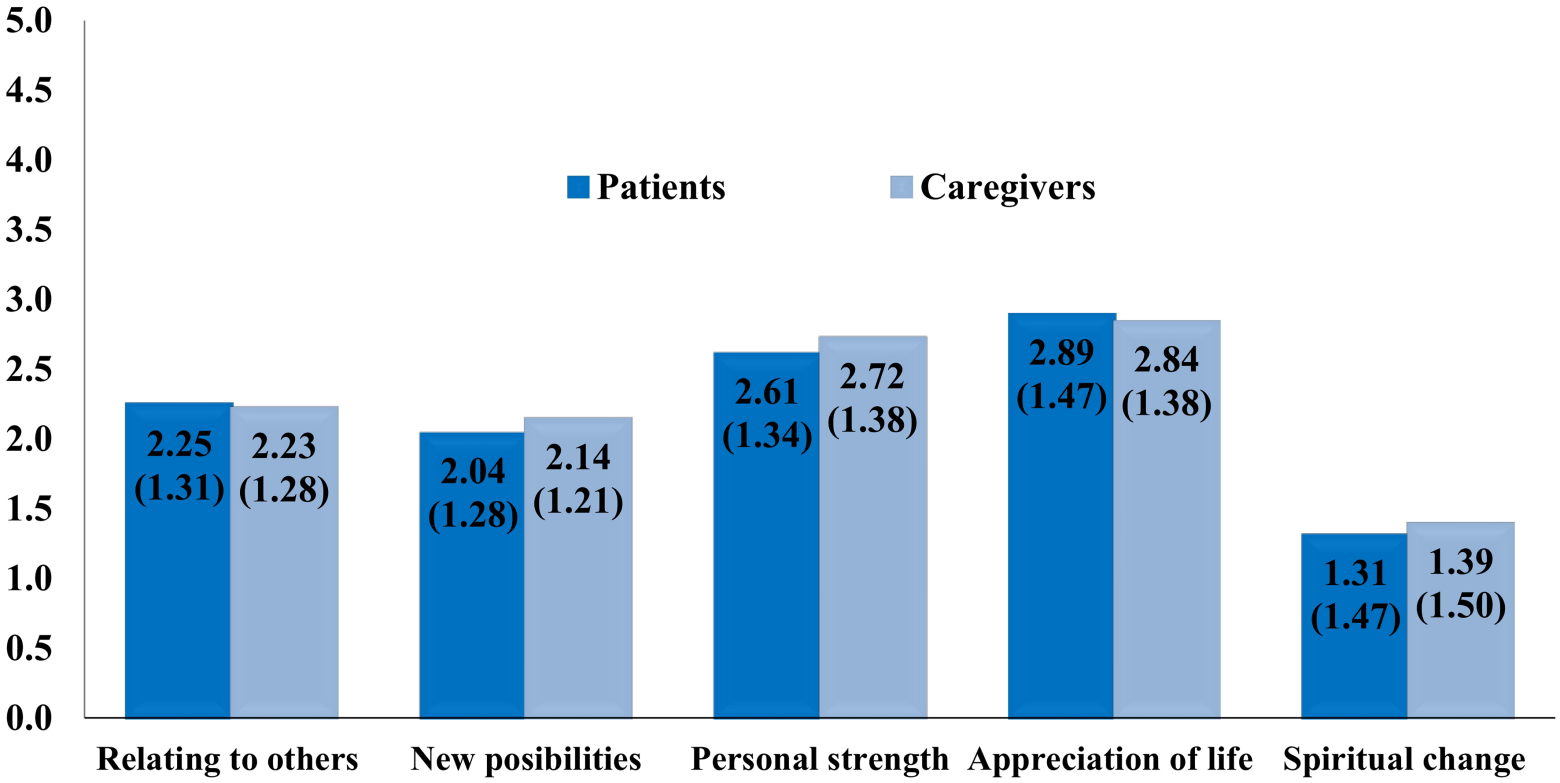
Mean scores (standard deviation) of patient and caregiver post-traumatic growth.

The comparison of patient and caregiver PTG1-21 subscale scores at T1 did not show statistically significant differences: Relating to others (*t* = 0.271, *p* = 0.79), New Possibilities (*t* = −0.925, *p* = 0.356), Personal Strength (*t* = −1.028, *p* = 0.305), Spiritual Change (*t* = 0.378, *p* = 0.378), Appreciation of Life (*t* = −0.884, *p* = 0.706) and Total Post-Traumatic Growth (*t* = −0.450, *p* = 0.653). Effect sizes were null (*d* from 0.015 to 0.081). Mean scores, standard deviation and paired t-test results are reported in Table 4 and Figure 3.

**Table 4:** Post-traumatic growth (PTGI-21) comparison between MS patients and caregivers (paired *t*-test).

	Patients	Caregivers	*t (p)*	Cohens’ *d*
	*M* (SD)	*M* (SD)		
Relating to others	2.25 (1.31)	2.23 (1.28)	0.271 (0.791)	0.015 (N)
New possibilities	2.04 (1.28)	2.14 (1.21)	−0.925 (0.356)	−0.081 (N)
Personal strength	2.61 (1.34)	2.72 (1.38)	−1.028 (0.305)	−0.081 (N)
Spiritual change	1.31 (1.47)	1.39 (1.50)	0.378 (0.378)	−0.054 (N)
Appreciation of life	2.89 (1.47)	2.84 (1.38)	−0.884 (0.706)	0.035 (N)
Total post-traumatic growth	47.68 (23.04)	48.51 (23.38)	−0.450 (0.653)	−0.036 (N)

N, Null effect size.

At T3 patients affected by COVID-19 (*n* = 123) compared to patients unaffected by COVID-19 (*n* = 137) did show significantly higher scores on the following scales: Relating to Others (*t* = 2.020, *p* = 0.044), New Possibilities (*t* = 2.868, *p* = 0.004), Spiritual Change (*t* = 3.699, *p* < 0.001), Appreciation of Life (*t* = 3.010, *p* = 0.002) and total Post-traumatic Growth (*t* = 3.188, *p* = 0.002). Effect sizes were small (*d* from 0.207 to 0.458). No significant differences were found between the COVID-19-affected group and the total sample (*d* from 0.110 to 0.303, null and small effect sizes), except for Spiritual Change (*t* = 2.131, *p* = 0.034, *d* = 0.303, small effect size). Results were similar when the unaffected group was compared with the total sample (*d* from 0.097 to 0.220, null and small effect sizes), the only significant difference was for Spiritual Change (*t* = 2.066, *p* = 0.039, *d* = 0.220, small effect size; Table 5).

**Table 5:** COVID impact at T3, mean scores and comparisons.

	*M* (SD)			Comparisons *p* (Cohen’s *d*)		
	A. COVID-affected (*n* = 123)	B. Not COVID-affected (*n* = 137)	C. Total sample (*n* = 260)	A-B	A-C	B-C
Relating to others	4.05 (0.92)	3.81 (0.99)	3.92 (0.97)	0.044 (0.251 S)	0.197 (0.137 N)	0.197 (0.112 N)
New possibilities	3.97 (1.08)	3.59 (1.05)	3.77 (1.08)	0.004 (0.357 S)	0.080 (0.182 N)	0.080 (0.169 N)
Personal strength	3.98 (1.12)	3.76 (1.00)	3.86 (1.06)	0.086 (0.207 S)	0.293 (0.110 N)	0.363 (0.097 N)
Spiritual change	2.28 (1.95)	1.42 (1.80)	1.83 (1.92)	<0.001 (0.458 S)	0.034 (0.303 S)	0.039 (0.220 S)
Appreciation of life	4.09 (1.08)	3.63 (1.38)	3.85 (1.24)	0.002 (0.371 S)	0.066 (0.206 S)	0.107 (0.160 N)
Total PTG	80.95 (18.85)	73.37 (19.42)	76.95 (19.49)	0.002 (0.396 S)	0.058 (0.208 S)	0.082 (0.184 N)

S, Small effect size; N, Null effect size.

## Discussion

In recent decades, there has been growing interest in positive changes derived from diagnosis and adaptation to MS. The first objective of this study was to analyze post-traumatic growth and its development in a large sample of MS patients.

Over the course of 36 months, there were significant increases in the total score and all the subscales except for spiritual change with large effect sizes. This confirms our first hypothesis. Less change in the Spiritual change subscale might be explained partly by methodological limitations of the questionnaire, as it is the only subscale consisting of just two items, and this limits its sensitivity to change. In addition, spiritual processes in particular might take longer to develop, exceeding the follow up period of 36 months. This is corroborated by the fact that even though the total increase on the spiritual subscale is less than on the others, the increase between T2 and T3 is more than threefold higher than the increase from T1 to T2. Spiritual Change is the most complex concept in the framework of post-traumatic growth, as it involves the definition of oneself in relation to the universe, which involves self, significant others, society, and any powers which may transcend and influence these concepts. In the two relevant items, the post-traumatic growth inventory specifically asks about “better understanding of spiritual matters” and “stronger religious faith.” A new orientation in this framework is highly challenging and requires time. Against this backdrop it is understandable that spirituality is usually stronger in older people (Malone and Dadswell, 2018). When comparing post-traumatic growth levels in the current study with previous observations in MS patients, it is important to consider the assessment time. Compared to previously reported levels, total post-traumatic scores were lower at T1 and T2, but higher at T3 (Aflakseir and Manafi, 2018; Kivi et al., 2019). At T2, total scores were higher than in patients with acquired brain damage (Rogan et al., 2013) or cardiac outpatients (Leung et al., 2010), at T3 higher than in cancer and myocardial infarction patients (Barskova and Oesterreich, 2009; Ackroyd et al., 2011). These findings are in keeping with previous results showing a higher level of post-traumatic growth in MS than other chronic diseases (Aflakseir and Manafi, 2018). Obviously, study design and the duration of follow up strongly influence the degree of post-traumatic growth reported. Regarding the specific pattern of post-traumatic growth in our study, the Appreciation of life and Personal strength dimensions were highest at T1. Contrary to this pattern, related research in MS (Kivi et al., 2019), cancer (Heidarzadeh et al., 2014), and myocardial infarction (Rahimi et al., 2016) has reported Spiritual change and Relating to others to be the most prominent post-traumatic growth dimensions. These differences might be explained by specific sociocultural circumstances of the patients in the samples favoring more individualistic approaches, such as in Western industrial societies or particularistic approaches as in Eastern societies.

In the conceptual foundation of post-traumatic growth by Tedeschi and Calhoun (2004), the successful management of distressing emotions is a prerequisite for cognitive processing and schema changes that might lead to post-traumatic growth. In this line of reasoning, persistent distress could be essential to developing the maximum level of post-traumatic growth. In MS patients, the need to deal with the variability of MS symptoms, chronicity, and unpredictable prognosis causes persistent distress. This continual coping could fuel cognitive processing and contribute to post-traumatic growth.

This study also analyzed possible factors influencing post-traumatic growth. Clinical variables, EDSS, pain interference and severity were predictors of post-traumatic growth at T2 and T3, respectively. Even though the vast majority of studies found no significant linear associations between disease severity and post-traumatic growth in either MS or other chronic conditions like acquired brain damage (Rogan et al., 2013) or cancer (Heidarzadeh et al., 2014), there is evidence that the subjectively felt magnitude of the trauma is an important variable, and in serious medical conditions, level of disability can determine its impact. Our result is in agreement with the study by Leung et al. (2010) relating post-traumatic growth to functional status in cardiac outpatients. Our study is the first to show a significant relationship between pain and post-traumatic growth in MS. Previous studies did not reveal these significant associations (Barskova and Oesterreich, 2009). We found pain severity to predict more post-traumatic growth, whereas interference predicted less. How can this seemingly contradictory finding be explained? Pain intensity mirrors the subjective perception of this negative sensation, which may induce coping strategies to manage or mitigate it. The deep psychological impact of a traumatic event is often described by affected individuals as painful, and therefore, pain intensity could even be considered a reminder of the traumatic stressor, and the ongoing traumatic pain fuels the process of adaptation and growth. As opposed to pain intensity, pain interference means the degree to which an individual is unable to manage the pain, possibly feeling overwhelmed and helpless. This reflects inability to cope with pain or actively deal with it and use it for personal growth. As neither time since diagnosis nor onset predicted post-traumatic growth, our second hypothesis could not be confirmed. This association was only found in one previous study of a sample of MS patients who had been diagnosed over 15 years before. In most other studies, no clinical or sociodemographic predictors of post-traumatic growth could be identified (Pakenham, 2005b; Hart et al., 2008; Pakenham and Cox, 2009; Prati and Pietrantoni, 2009; Ackroyd et al., 2011). Interestingly, significant prediction of post-traumatic growth by time since diagnosis or transplantation could not be confirmed in either cancer (Heidarzadeh et al., 2014) or liver transplant patients (Pérez-San-Gregorio et al., 2017b). In other words, in none of these empirical studies did the exact time since diagnosis predict the inner process of post-traumatic growth. This might indicate that the psychological process is driven by highly complex cognitions and does not necessarily develop in a continuously ongoing stream, but rather by long unforeseeable phases of rumination and sudden life-changing insights. Therefore, these idiosyncratic processes reflect a highly individual inner time flow. Tedeschi and Calhoun (2004) themselves emphasized the nonlinearity of post-traumatic growth and proposed, for example, a complex association curve between trauma strength and post-traumatic growth.

Accordingly, the underlying mechanisms of post-traumatic growth are closely related to cognitive factors, which lead to the restructuring of personal narratives. In this context the presence of deliberate versus intrusive cognitions as well as frequency, timing, valence and content of cognitions need to be taken into account (Tedeschi and Calhoun, 2004).

By demographic factors, female gender and lower education were predictors of higher post-traumatic growth at T2. Previous studies have consistently shown a close association between female gender and post-traumatic growth (Henson et al., 2021). In this context, the tendency of women to deliberately ruminate on constructive issues, such as personal strengths or the importance of social connections, has been suggested as a relevant mechanism (Vishnevsky et al., 2010). This cognitive process is facilitated by the stronger tendency of women to share their experiences with others (Henson et al., 2021). This finding is supported by previous research in cancer and HIV survivors (Barskova and Oesterreich, 2009). Moreover, lower education was a positive predictor of post-traumatic growth. As mentioned above, there are several studies demonstrating that individuals with less education tend to rely more on others and use the resources of social relationships, which may be a crucial resource in fostering growth from adversity (Heidarzadeh et al., 2014; Henson et al., 2021).

Of the mental health variables, greater social dysfunction predicted lower levels of post-traumatic growth at T1 and T2, and greater anxiety and insomnia higher post-traumatic growth at T1. It has consistently been shown that sharing emotions as well as disclosure of the traumatic event in a safe social environment is helpful for post-traumatic growth development. Additionally, talking to others indirectly promotes post-traumatic growth by reducing depressive symptoms (Prati and Pietrantoni, 2009; Leung et al., 2010; Henson et al., 2021). On the contrary, difficulties in relating to others and sharing, as in social dysfunction, can hamper progress of post-traumatic growth.

Anxiety and insomnia positively predicted post-traumatic growth at T1. Anxiety, as opposed to depression, is associated with more of a sympathetic tone, which may induce active coping mechanisms to handle the stressor and reduce the stress reaction. A similar association between post-traumatic growth and anxiety has previously been reported in MS patients as well as in acquired brain injury survivors (Barskova and Oesterreich, 2009).

In an attempt to find external/circumstantial variables that could explain post-traumatic growth in MS, we studied the differences between patients who had experienced that the COVID pandemic had impacted on their lives and those who had not. The COVID-19-affected group showed higher post-traumatic growth levels than the unaffected group in the final evaluation (T3). The onset of the pandemic might be a further significant life event, experienced as a significant stressor and adding to the impact of highly personal life events like MS, thereby increasing post-traumatic growth. However, as the pandemic began near the end of the study, its impact on overall changes in post-traumatic growth should not be overestimated. Apparently, objective clinical parameters, such as EDSS, MS type, disease duration, and symptomatology do not directly influence post-traumatic growth, but rather, their subjectively felt impact and intrapsychic coping strategies in dealing with them. Active coping, such as positive reframing, acceptance, and seeking emotional and social support could lead to higher post-traumatic growth than passive strategies such as avoidance and denial. Therefore, further research on post-traumatic growth in MS coping strategies should be considered essential.

Based on our findings the underlying mechanisms in post-traumatic growth involve the ongoing intrapsychic impact of a traumatic event, which manifests in pain, anxiety, or disability, which, however, does not substantially hamper social interactions but rather strengthens social relationships. Therefore, social dysfunction, pain interference and male gender are factors contributing to significantly less growth.

Our third hypothesis could not be confirmed, as there were no significant differences at T1 between patient and caregiver total post-traumatic scores or subscales. In previous studies where time since diagnosis was comparable to our study (about 10 years vs. 12 years in our study) significantly higher post-traumatic growth was reported in MS patients than in caregivers (Pakenham, 2005b; Ackroyd et al., 2011). A similar outcome was found after liver transplantation (Pérez-San-Gregorio et al., 2017b).

A possible explanation for these differences could be that in our sample disability was lower with an EDSS score of 3.7, indicating that patients were still able to walk without issues, compared to 5.1 in the study by Ackroyd et al. (2011), meaning the disability was severe enough to impair full daily activities and ability to work a full day without special provisions and patients were only able to walk without aid or rest for 200 m. The other study (Pakenham, 2005b) did not provide an EDSS score.

Nevertheless, our study corroborated empirical findings insofar as MS led to post-traumatic growth not only in patients but also in caregivers. Recent research has highlighted the importance of open communication about personal experiences, creating a “communal sense of the illness” which fosters growth and personal gain in the MS patient-caregiver dyad (Neate et al., 2018, 2019; Treder-Rochna, 2020). By means of empathy and charitable understanding, caregivers delve into the patient’s inner world, fueling cognitive changes in their own. This might be easier when the caregiver is the partner, which was the case in 65% of our sample. The fruitful connection of otherwise distinct inner worlds may be a good example of “shared pain is of half the pain.” Future research could find out whether patient-caregiver post-traumatic growth patterns still match over a 36-month longitudinal study.

### Implications for Clinical Practice

Our study confirmed significant post-traumatic growth in MS even an average 12 years from diagnosis even without any psychotherapeutic help to support or enhance this development. Nevertheless, as stronger pain interference and social dysfunction, as well as higher education and male gender were associated with less growth, it would be important to optimize post-traumatic growth in patients with these characteristics. In clinical practice, cognitive behavioral therapy has been shown to support post-traumatic growth (Knaevelsrud et al., 2010). Cognitive behavioral therapy is the most empirically validated form of psychotherapy and has undergone remarkable development categorized in three phases or waves. The evolution started with behavioral therapy, in the second wave, cognitive influences on behavior were addressed in cognitive therapy, and in the third wave, acceptance-based therapies focused on mindfulness and compassion. Acceptance and Commitment Therapy (ACT), Dialectic Behavioral Therapy (DBT) and Mindfulness-Based Stress Reduction (MBSR) as well as mindfulness-based cognitive therapy (MBCT) belong to the so-called third wave. The essence of Acceptance and Commitment Therapy (ACT) is that it helps the individual find important values and goals for directing behavioral change. Dialectic Behavioral Therapy (CBT) aims at emotion regulation, distress tolerance, self-acceptance, and validation. It can be helpful for patients whose personality structure is less well-integrated and for comorbid personality disorder. Finally, Mindfulness-Based Stress Reduction (MBSR), and Mindfulness-Based Cognitive Therapy (MBCT), including components of CBT, focus on awareness of the present moment and acceptance (Zarotti et al., 2022). All these therapies help patients to stay focused on the present moment and accept thoughts and feelings without judgment, which might be particularly helpful in patients suffering from pain interference or social dysfunction, as by accepting their situation, patients can acquire more control and feel less helpless, similar to what has been demonstrated for similarly traumatic diseases (Eccles et al., 2021). Acceptance and creating meaning from a stressful situation, reframing the life-impacting event, validating the personal experience, appreciating life and redefining life goals are psychological mechanisms involved in an MS patients’ post-traumatic growth that can be supported by third-wave CBT intervention.

### Limitations and Strength

The main weakness of the study was non-random sampling at a single tertiary care center, which limits its external validity. In addition, the use of self-report questionnaires increases the risk of bias due to social desirability. Evaluation of mental health with a semi-structured interview, for instance, could be more reliable. Nonetheless, the large sample size and low dropout rate, its heterogeneity and longitudinal design over 36 months are major strengths of this trial.

## Conclusion

Our results suggest significant positive consequences for MS patients over a 36-month follow-up, even 12 years from diagnosis. Higher EDSS, more severe pain, less pain interference, female gender, lower education, higher anxiety and lower social dysfunction showed positive linear associations with post-traumatic growth. These results highlight the importance of a thorough assessment of a wide spectrum of sociodemographic and clinical variables leading to further knowledge of the underlying mechanisms of post-traumatic growth in MS.

Comparable levels of post-traumatic growth in MS patients and caregivers provide insight into possibilities for personal growth driven by care and empathy, which can be seen as an inspiring and hopeful message.

## Data Availability Statement

The raw data supporting the conclusions of this article will be made available by the authors, without undue reservation.

## Ethics Statement

The studies involving human participants were reviewed and approved by Ethical Committee of Virgen Macarena University Hospital (0846-N-18). The patients/participants provided their written informed consent to participate in this study.

## Author Contributions

IG-G, MÁP-S-G, RC, and AM-R contributed to conception and design of the study, wrote sections of the manuscript, organized the database, performed the statistical analysis, and wrote the first draft of the manuscript. All authors contributed to manuscript revision, read, and approved the submitted version.

## Funding

This study was funded by a grant from the Spanish Ministry of Education, Culture and Sports under University Professor Training Program Grant no. FPU 17/04240 and co-funded by the European Regional Development Fund (ERDF) and the Regional Ministry for Economy Transformation, Industry, Knowledge, and Universities of the Junta de Andalucía [Andalusian Regional Govt.], under Operational Program ERDF 2014–2020 (reference: US-1379382).

## Conflict of Interest

The authors declare that the research was conducted in the absence of any commercial or financial relationships that could be construed as a potential conflict of interest.

## Publisher’s Note

All claims expressed in this article are solely those of the authors and do not necessarily represent those of their affiliated organizations, or those of the publisher, the editors and the reviewers. Any product that may be evaluated in this article, or claim that may be made by its manufacturer, is not guaranteed or endorsed by the publisher.
